# Lymphatic vessel density and function in experimental bladder cancer

**DOI:** 10.1186/1471-2407-7-219

**Published:** 2007-11-29

**Authors:** Marcia R Saban, Rheal Towner, Nataliya Smith, Andrew Abbott, Michal Neeman, Carole A Davis, Cindy Simpson, Julie Maier, Sylvie Mémet, Xue-Ru Wu, Ricardo Saban

**Affiliations:** 1 Department of Physiology, College of Medicine, Oklahoma University Health Sciences Center (OUHSC), Oklahoma City, OK 73104, USA; 2 Small Animal MRI Core Facility, Free Radical Biology & Aging, Oklahoma Medical Research Foundation (OMRF), Oklahoma City, OK 73104, USA; 3 Department of Biological Regulation, Weizmann Institute of Science, Rehovot, 76100, Israel; 4 Oklahoma Medical Research Foundation (OMRF), Imaging Core Facility, Oklahoma City, Oklahoma 73104, USA; 5 Unité de Mycologie Moléculaire, URA CNRS 3012, Institut Pasteur, 75724 Paris Cedex 15, France; 6 Department of Urology, New York University Medical School, New York, NY 10016, USA

## Abstract

**Background:**

The lymphatics form a second circulatory system that drains the extracellular fluid and proteins from the tumor microenvironment, and provides an exclusive environment in which immune cells interact and respond to foreign antigen. Both cancer and inflammation are known to induce lymphangiogenesis. However, little is known about bladder lymphatic vessels and their involvement in cancer formation and progression.

**Methods:**

A double transgenic mouse model was generated by crossing a bladder cancer-induced transgenic, in which SV40 large T antigen was under the control of uroplakin II promoter, with another transgenic mouse harboring a *lacZ *reporter gene under the control of an NF-κB-responsive promoter (κB-*lacZ*) exhibiting constitutive activity of β-galactosidase in lymphatic endothelial cells. In this new mouse model (SV40-*lacZ*), we examined the lymphatic vessel density (LVD) and function (LVF) during bladder cancer progression. LVD was performed in bladder whole mounts and cross-sections by fluorescent immunohistochemistry (IHC) using LYVE-1 antibody. LVF was assessed by real-time *in vivo *imaging techniques using a contrast agent (biotin-BSA-Gd-DTPA-Cy5.5; Gd-Cy5.5) suitable for both magnetic resonance imaging (MRI) and near infrared fluorescence (NIRF). In addition, IHC of Cy5.5 was used for time-course analysis of co-localization of Gd-Cy5.5 with LYVE-1-positive lymphatics and CD31-positive blood vessels.

**Results:**

SV40-*lacZ *mice develop bladder cancer and permitted visualization of lymphatics. A significant increase in LVD was found concomitantly with bladder cancer progression. Double labeling of the bladder cross-sections with LYVE-1 and Ki-67 antibodies indicated cancer-induced lymphangiogenesis. MRI detected mouse bladder cancer, as early as 4 months, and permitted to follow tumor sizes during cancer progression. Using Gd-Cy5.5 as a contrast agent for MRI-guided lymphangiography, we determined a possible reduction of lymphatic flow within the tumoral area. In addition, NIRF studies of Gd-Cy5.5 confirmed its temporal distribution between CD31-positive blood vessels and LYVE-1 positive lymphatic vessels.

**Conclusion:**

SV40-*lacZ *mice permit the visualization of lymphatics during bladder cancer progression. Gd-Cy5.5, as a double contrast agent for NIRF and MRI, permits to quantify delivery, transport rates, and volumes of macromolecular fluid flow through the interstitial-lymphatic continuum. Our results open the path for the study of lymphatic activity *in vivo *and in real time, and support the role of lymphangiogenesis during bladder cancer progression.

## Background

*De novo *lymphangiogenesis influences different pathological courses via modulating tissue fluid homeostasis, macromolecule absorption, and leukocyte transmigration [1]. In addition, lymphatic vessels play a crucial role in a variety of human cancers [2]. Invasion of lymphatic vessels by tumor cells and subsequent development of lymph node metastases significantly influences the prognosis of cancer patients and, therefore, represents an integral part of tumor staging. Increasing knowledge of the tumor's biological significance in lymphatics within the tumors and at the tumor periphery has greatly promoted understanding of tumor access into the lymphatic system by inducing lymphangiogenesis or by co-opting preexisting lymphatics [2]. In contrast, impaired functioning of lymphatic vessels results in lymphedema as observed during breast cancer diagnosis and treatment [3-5].

During cancer progression, a bi-directional communication is established between the tumor microenvironment (TME) and lymphatic vessels. In one direction, the lymphatic vasculature alters TME by draining the interstitial protein-rich exudate fluid (lymph) into the bloodstream. In another direction, inflammation influences the composition and pressure of TME leading to altered lymphatic vessel function.

We choose to study bladder cancer because it represents 2% of all human malignancies. Urothelial carcinoma is one of the most common cancers – it ranks fifth among all cancers in the Western world, and there are 336,000 new cases and 132,000 deaths annually worldwide [6]. In the US alone, the American Cancer Society estimates that 50,040 men and 17,120 women will be diagnosed, and 13,060 men and women will die of cancer of the urinary bladder in 2007 [7]. Although the role of lymphatic vessels during bladder cancer progression is remarkably unknown, invasion of lymphatics during bladder cancer has been reported [8], whereas in prostate cancer there is a decrease in intratumoral lymphatic vessel density [9]. More recently, Fernandez and collaborators published the first manuscript suggesting the existence of proliferating lymph vessels and, therefore, of lymphangiogenesis in bladder transitional cell carcinoma (TCC), and proposed strong correlation of higher peritumoral LVD with the presence of lymph nodes in clinically localized invasive bladder TCC [10]. However, up to now, no animal model was available for a systematic study of lymphatic vessel density and function during bladder cancer progression.

We previously described that a transgenic mouse (κB-*lacZ*) with a reporter gene (*lacZ*) for NF-κB presented constitutive β-galactosidase (β-gal) activity in all lymphatic endothelial cells [11] and that these mice serve a dual purpose by permitting both visualization of lymphatics and detection of constitutive and inducible NF-κB activity [11]. To study in-depth the role of lymphatic vessels in bladder cancer progression, we generated a double transgenic mouse (*SV40-lacZ*) by crossing the κB-*lacZ *mice with a well established model of bladder cancer (UPKII/SV40T) [12,13]. Here, we demonstrate that these SV40-lacZ mice present an increased lymphatic vessel density during bladder cancer progression.

We also show that a new compound, coined Gd-Cy5.5, which corresponds to a recently described contrast agent (biotin-BSA-Gd-DTPA) [14-16] conjugated to Cy5.5 permits dual imaging by near-infrared fluorescent (NIRF) and MRI. We, therefore, provide proof-of-concept that this contrast agent can be used for determination of lymphatic vessel function during bladder cancer progression.

## Methods

### Animals

All experiments were performed according to the "Principles for Research Involving Animals and Human Beings Guidelines" (OUHSC Animal Care & Use Committee protocol # 04–028). Double transgenic mice were obtained by crossing κB-*lacZ *with UPKII/SV40T mice. The κB-*lacZ *transgenic model used in this study was first described in 1996 [17] and enriched in the C57Bl/6 background. It was constructed using the promoter of the gene encoding p105, a precursor of the p50 subunit of NF-κB. This promoter contains three NF-κB binding sites in its proximal part driving the expression of *lacZ *with a nuclear localization sequence. These mice permit the visualization of lymphatic endothelial cells in the urinary bladder and all other tissues examined so far [17].

A double transgenic mouse model (*SV40-lacZ*) was generated by crossing the κB-*lacZ *transgenics with UPKII/SV40T mice. UPKII/SV40T mice present the SV40 large T antigen under the control of a urothelium-specific mouse uroplakin II promoter, and develop specifically bladder cancer [12,18,19]. The phenotype of each mouse was confirmed by southern blot analysis of SV40T and PCR for β-galactosidase in tail vein snips. Only double positive mice were used in these experiments. FVB mice were purchased from Jackson Labs, crossed with κB-*lacZ*, and used as controls for UPKII/SV40T mice.

### Lymphatic Vessel Density (LVD)

We follow the "Reporting Recommendations for tumor Marker (REMARK)" guidelines [20]. This consensus report aims to lower the methodological variability of lymphangiogenesis quantification in tissue sections. The recommendations include: **1) **double-blinded experiments; **2) **the use of multiple IHC stains on serial sections (We used LYVE-1 IHC that has been established in our laboratories [11] and X-gal staining for β-galactosidase, because the SV40-*lacZ *enable this stain for visualization of lymphatic vessels [11]); and **3) **the use of a double immunostaining of lymphatic marker and those for cell proliferation. In this context, bladder cross-sections were double stained with LYVE-1 and Ki-67 antibodies.

### LVD in bladder whole-mounts

LVD was determined as described before [11,17]. Eight mice per point were euthanized with sodium pentobarbital (100 mg/kg, i.p.) and tissues were removed rapidly and stained as whole mounts with X-gal. β-galactosidase activity was revealed by X-gal staining at 30°C for 4–6 hours. Whole mounts were examined under a dissecting scope (SMZ 1500, Nikon). All tissues were photographed by a digital camera (DXM1200; Nikon). Exposure times were held constant when acquiring images from different tissues. Afterwards, tissues were washed four times in PBS and post-fixed in 2% paraformaldehyde in PIPES [piperazine diethanesulfonic acid]. Lymphatic vessel density was quantified by morphometric analysis using Neurolucida workstation (MicroBrightField, Inc) [21], as described (ref [11]; supplemental material). Morphometric analysis was performed by importing Neurolucida tracings into NeuroExplorer software version 3.70.2 (MicroBrightField, Inc) [21]. The ratio between the area occupied by lymphatics and the total tissue area (μm^2^) was then determined for each section and statistical analysis was performed using a Wilcoxon's rank sum test. Results are expressed as mean ± SEM. In all cases, a value of p < 0.05 was considered indicative of a significant difference [22]. This method may result in the overestimation of the lymphatic area. The software uses the 3D surface area of lymphatics, estimated from a 2D projection. Also, tissue area was calculated from a 2D projected cross-sectional tissue area (tissue contour).

### Fluorescence IHC

Frozen bladders were processed for routine IHC according to published methods [23]. Frozen sections were post-fixed in 1% formaldehyde. All reagent incubations and washes were performed at room temperature. 5% normal donkey blocking serum (Jackson Immunolabs) was placed on all slides for 45 min and sections were incubated with primary antibody for 1 hour and 45 minutes in a humidifier chamber. Slides were washed 3× 5 minutes in PBS and incubated with secondary antibodies. Slides were washed, counterstained with DAPI [4',6-diamidino-2-phenylindole; 1:20,000 dilutions of 10 mg/ml] for 2 minutes and coverslipped with Shur/Mount (TBS) mounting media and sealed with nail polish. All tissue cross-sections were visualized with a Nikon Eclipse TE 2000-S inverted fluorescent microscope (Nikon) [24] and imaged at room temperature using a digital CCD camera (Roper Scientific) [25], driven by NIS-Elements AR2.3 Imaging software (Laboratory Imaging/Nikon) [26]. Controls included omission of the primary antibody. Antibodies used in this work are listed in Table 1. All secondary antibodies were used at a 1:400 dilution. Secondary antibodies included donkey anti-rabbit IgG AF488 conjugate (Molecular Probes; probes.invitrogen.com), donkey anti-goat IgG (Alexa Fluor 546, A11056, Invitrogen), and donkey anti-rat IgG (AlexaFluore 488).

**Table 1:** Primary antibody characteristics

*Antigen*	*Host Species*	*Against*	*Dilution*	*Code*	*Supplier*
CD31	Rat	Mouse	1:50	550274	Abcam [67]
LYVE-1	Rabbit	Mouse	1:400	ab14917	Abcam [67]
Ki-67	Goat	Mouse	1:500	sc-7846	Santa Cruz [68]
MAC 387 (calprotein Ab-1) monoclonal	Mouse	Mouse	1:1000	Mac 387	Lab Vision [69]

### LVD and image analysis of bladder cross-sections

At least 6 random fields per cross-section were visualized at 20× magnification and used for image analysis that was performed with the NIS-Elements Advanced Research 2.3 imaging software [26]. This software identifies signal by thresholding key intensity values. Further the software permits imposing restrictions to the measurements by excluding false positive signals. Briefly, the number of positive cells expressing a particular antibody was calculated as percent of the region of interest (ROI), as indicated in the individual figure legend. Co-localization of two antibodies was calculated by converting the area occupied by cells positive for the first antibody into a ROI. Then the percent of cells that were positive for the second antibody was calculated within the ROI. Results are expressed as mean ± SEM. In all cases, a value of p < 0.05 was considered indicative of a significant difference [22].

### NIRF

Mice were fed a low-chlorophyll diet for 2 weeks to reduce auto-fluorescence in the intestinal region [27] and the abdominal hair was removed. Mice were anesthetized with isoflurane, placed in a heating pad, and received 200 μL of the Gd-Cy5.5 intravenously, and the accumulation of Cy5.5 into the urinary bladder was followed over time. Anesthetized mice were immediately placed on a heating pad inside a FluorChem HD2 (Alpha Innotech, San Leandro, CA) equipped with a Chromalight^® ^multi-wavelength illuminator and a 4-million pixel Cooled camera (F2.8 manual zoon lens and F1.4 fixed lens) coupled to a dedicated computer. The FluorChem cabinet permits continued anesthesia with isoflurane. Images were first acquired and stored with AlphaEase FC^® ^32-bit software (Alpha Innotech, San Leandro, CA) and subsequently, application of black-and-white and color gradients were performed in Adobe Photoshop^® ^CS3 extended [28] that permitted the determination of integrated density. For this purpose, an elliptical marquee of fixed size (180 × 180 px) was used to determine the region of interest (ROI) around the luminescence zones corresponding to a bladder area and the count tool was used to determine and record each integrated density. The integrated density corresponded to the sum of the values of the pixels in the ROI, which were equivalent to the product of the area (in pixels) and mean gray value.

### MRI

All data was acquired using a Bruker 7 Tesla/30 cm horizontal bore magnet. Anatomical scans acquired proved to be useful in elucidating normal and bladder tumor tissue. Bladder and tumor physiology was followed in a time course study using this method at each time point, for each animal. This method is a dual spin-echo technique modified from a MSME (Multi-Slice Multi-Echo) technique designed to yield anatomical T1 and T2 weighted images for each slice position in the slice package. Axial scans acquired had the following parameters: TR = 900 ms, TE = 11.6 ms, slice thickness = 0.75 mm, NA = 4, slice gap = 0.05 mm, FOV = 2.5 cm × 2.5 cm, matrix size = 256 × 256, giving an in-plane resolution of 98 μm × 98 μm. T1-weighted images had an effective TE of 17 ms, and T2-weighted images had an effective TE of 52 ms. Acquired parameter images had TE = 15 ms over a range of 5 TR increments with values = (100, 300, 450, 600, 850, and 1150 ms), slice thickness = 0.75 mm (position of slice varied according to tumor location), FOV = 2.5 cm × 2.5 cm, NA = 2, matrix size = 128 × 128, and an in-plane resolution of 195 μm × 195 μm. Mono-exponential fits were utilized to calculate actual T1 values in post-processing.

### Synthesis of biotin-BSA-GdDTPA compound

The macromolecular contrast material, biotin-BSA-GdDTPA, was prepared by the modification of the method of Dafni and collaborators [29]. Bovine serum albumin (BSA, 0.5 g, 8 μmol; Sigma) was dissolved in 0.1 M sodium bicarbonate (7.5 ml, pH 8.5). Sulfo-NHS-Biotin (22.4 mg; 53 μmol; Pierce) was dissolved in double distilled water (DDW, 1.2 ml) and was added to BSA while stirring. The reaction mixture was stirred for 1 hr at 4°C and an additional 2 hrs at room temperature. The dialyzed product in 0.1 M Hepes buffer (pH 8.8) was reacted with diethylene triamine pentaacetic acid anhydride (DTPA, 0.5 g, 1.4 mmol; Sigma) suspended in 2.5 ml of dimethyl sulfoxide (DMSO) at room temperature. DTPA was added in portions and the pH was adjusted immediately after each addition to 8.5 with 5 N NaOH. The reaction mixture was stirred for 2 hrs at 4°C and extensively dialyzed against cold 0.1 M citrate buffer (pH 6.5). Finally, gadolinium (III) chloride (GdCl_3_, 0.25 g; 0.67 mmol; Sigma) in 2.5 ml 0.1 M sodium acetate buffer (pH 6.0) was added gradually, and the mixture was stirred for 24 hrs at 4°C. The product, biotin-BSA-GdDTPA, was extensively dialyzed against cold citrate buffer (0.1 M, pH 6.5) and then against DDW. The product was lyophilized and stored at 4°C. For Cy5.5 -Gd preparation, 10 mg of dry lyophilized product (biotin-BSA-GdDTPA) was reconstituted in 150 μl of sodium bicarbonate buffer 0.1 M pH 8.8 NaHCO_3_. Cy5.5 dye (Cy5.5 mono functional reactive dye, Amersham Biosciences) was dissolved in 10–20 μl of DMF and added to the product. The mixture was incubated in the dark at RT for 1 hr while mixing. The final compound biotin-BSA-GdDTPA-Cy5.5 was purified using Zeba 2 ml columns and had a molecular weight of ~82 kDa.

### Other compounds

Dextran (500,000 MW)-Cy5.5 (Dex 0003–5) was purchased from Nanocs [30].

## Results

### Visualization of bladder lymphatics

**Figure 1:**
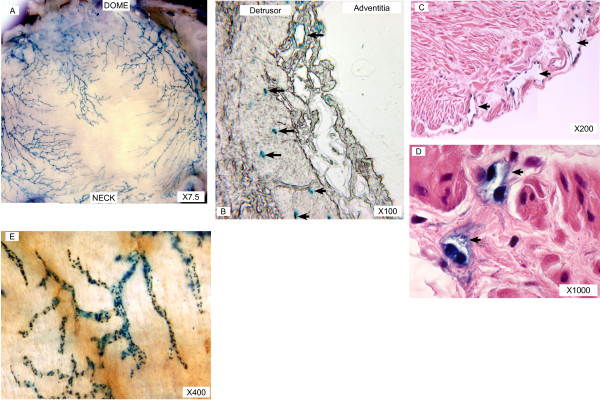
**A-E. Visualization of lymphatic vessels in a double transgenic mouse model**. SV40-*lacZ *mice obtained by crossing κB-*lacZ *mice with UPKII/SV40 maintain the capacity of the parent mice in developing bladder carcinoma *in situ *(CIS) and labeling of bladder lymphatic vessels. **A **represents X-Gal staining of bladder whole mounts isolated from SV40-*lacZ *mice, indicating a rich network of lymphatic vessels in the bladder adventitia. Cross-sections of the urinary bladder illustrated in **A **were left unstained **(B) **or counter stained with H&E **(C-D)**. Figure **C **is a representative of bladder adventitia and **D **is a high magnification of lymphatic vessels present in the detrusor smooth muscle. Arrows (**B, C, and D**) indicate the presence of β-galactosidase-positive lymphatic endothelial cells. **E **is a representative whole mount of bladders isolated from SV40-*lacZ *mice subsequently stained with X-gal to reveal β-galactosidase-positive cells (blue) and LYVE-1 IHC to reveal lymphatic endothelial cells (brown). Note the complete overlap of labels confirming constitutive NF-κB activity in lymphatic vessels.

**Figure 2:**
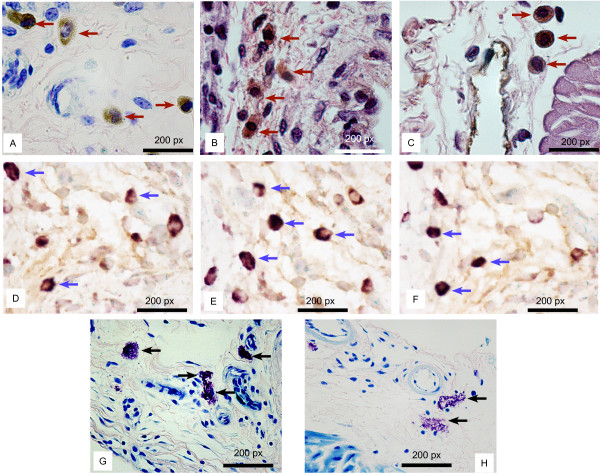
**Presence of LYVE-1-positive macrophages during bladder cancer progression**. Representative photomicrographs indicating LYVE-1-positive cells resembling macrophages (red arrows) in the urinary bladders obtained from SV40-*lacZ *mice. **A **= LYVE-1 and Giemsa stain. **B **and **C **= LYVE-1 and H&E stained cross-sections. **D, E, and F = **MAC 387 immunohistochemistry (blue arrows) performed in serial sections of the same bladders represented in **A, B, **and **C**. Mast cells (black arrows) present in the cross-sections were positive for Giemsa stain, but negative for LYVE-1 (**D **and **E**).

As the parent mouse model (κB-*lacZ*) permitted the visualization of lymphatic vessels by the expression of β-galactosidase, we investigated whether the double transgenic maintained this capacity. Figure 1A is a representative whole mount X-gal staining of bladders isolated from SV40-*lacZ *indicating that these mice maintained the constitutive activity of NF-κB in lymphatic vessels. Positive X-gal staining was also observed in cross sections left unstained (Figure 1B) or counter stained with H&E (Figure 1C and 1D). High magnification pictures of bladder cross sections indicate a nuclear localization of β-galactosidase expression in lymphatic endothelial cells due to a nuclear localization sequence of the original construct of the transgenic mouse (Figure 1D). Double staining of whole mounts confirmed that all β-galactosidase positive vessels were also LYVE-1-positive, and therefore, were lymphatic vessels (Figure 1E). In addition to lymphatic endothelial cells, cells morphologically resembling macrophages were the only other cell type that also showed LYVE-1 staining (Figures 2 A–C). This was further supported by the fact that these macrophage-like cells expressed MAC3, a macrophage marker [31] (Figures 2D–F). In contrast, other inflammatory cells were negative for LYVE-1 IHC. This is the case of mast cells present in the mouse bladder (Figure 2G–H).

### Increased lymphatic vessel density (LVD) during mouse BC progression

**Figure 3:**
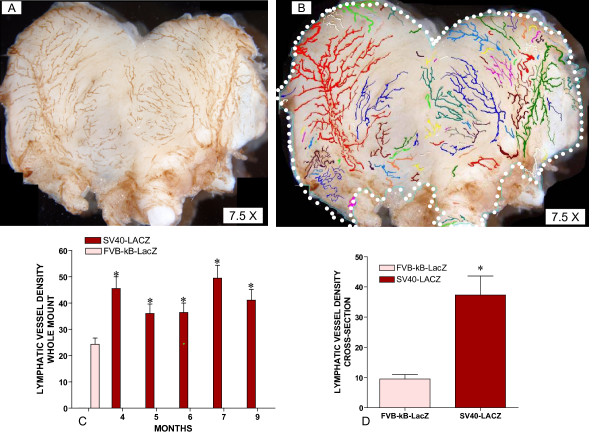
**A-D. Increased lymphatic vessel density during bladder cancer progression**. LYVE-1 IHC quantification of lymphatic vessel density (LVD) in bladders isolated from control and SV40-*lacZ *mice. **A = **Representative photomicrographs of LYVE-1 IHC. **B = **Neurolucida tracing of lymphatics. **C = **Lymphatic vessel density during cancer progression in whole mount preparations of LYVE-1 stained bladders isolated from SV40-*lacZ *mice compared to control (FVB-κB-*lacZ *= pink). **D = **Lymphatic vessel density during cancer progression in cross-section preparations of LYVE-1 stained bladders isolated from SV40-*lacZ*mice compared to control (FVB-κB-*lacZ *= pink). Asterisks indicate a statistical significant difference (p < 0.05) between FVB-C57BL/6 and SV40-*lacZ *mice.

Lymphatic vessel density was determined in control (FVB-κB-*lacZ*) and SV40-*lacZ *mice by Neurolucida^® ^guided morphological analysis of LYVE-1 IHC stained bladder whole mounts, as described (supplemental material to manuscript [11]) and in cross-sections by fluorescent IHC. Figure 3A and 3B are representative LYVE-1 IHC of a urinary bladder isolated from SV40-*lacZ *mice. LYVE-1 staining was correlated with β-gal expression leading to results similar to those presented in Figure 1E and, therefore, indicating that the vessels represented in Figure 3A are indeed lymphatics. After IHC with LYVE-1 (Figure 3A) Neurolucida^® ^was used to trace and quantify the region of interest (ROI = white dotted line) and volume of lymphatic vessels (Figure 3B). Figure 3C presents the lymphatic vessel density (percent per unit of area) indicating that compared to 6-months old FVB-κB-*lacZ *mouse (n = 12), SV40-*lacZ *mice (n = 4 for each time point) presented a significant increase in LVD in the adventitia during cancer progression (BC = mean LVD 39.3% (n = 20); and control (FVB-κB-*lacZ *mice) = mean LVD 24.3%, [n = 12]; p < 0.001). This increase in LVD was observed in as young as 4-month old mice and remained elevated in all age groups studied so far (up to 9 months). Because LVD in whole mounts could only take into consideration medium and large lymphatics, an additional group of mice had their bladders removed and their cross-sections were studied by fluorescent IHC using LYVE-1 antibody. Image analysis of the cross-sections of 9-month old FVB-κB-*lacZ *and SV40-*lacZ *mice (ages 7, 9, and 11 months) are presented in Figure 3D (a total of 11 control and 12 SV40-*lacZ *cross-sections were examined), confirming a significant increase in LVD during bladder cancer progression.

### Bladder cancer-induced lymphangiogenesis

**Figure 4:**
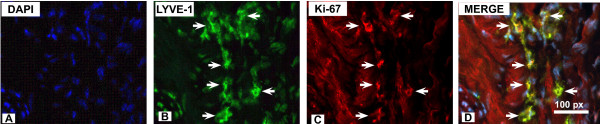
**Bladder cancer-induced lymphangiogenesis**. Next, the same cross- sections illustrated in **Figure 3 **were double-labeled with antibodies against LYVE-1 and a marker of cell proliferation, Ki-67. **A-D **are representative photomicrographs of the sub-urothelial lymphatic vessels illustrating the finding that some of the lymphatic endothelial cells were positive for both LYVE-1 (green fluorescence in **B**) and cell proliferation (Ki-67-positive cells are in red fluorescence in **C**), as illustrated by a yellow labeling of merged images in **D**. These results indicate the presence of cancer-induced lymphangiogenesis in this mouse model. Blue stain in **A **is DAPI (4',6-diamidino-2-phenylindole) which highlights the cell *nuclei*.

Next, the same cross- sections used for quantification of LYVE-1 were double labeled with LYVE-1 and an anti-goat polyclonal antibody against Ki-67(M-19), a nuclear protein expressed in proliferating cells that may be required for maintaining cell proliferation [32,33], and that provides the same value for proliferation index as BrdU in urothelial cancer cells [33]. Figure 4A–D is a representative photomicrograph of sub-urothelial lymphatic vessels illustrating the finding that some of the lymphatic endothelial cells were positive for both LYVE-1 and Ki-67. These results indicate the presence of cancer-induced lymphangiogenesis in this mouse model.

### Lymphatic Vessel Function (LVF) by NIRF

For NIRF, a total of 9 SV40-*lacZ *mice ages 6–11 months were used. Mice were fed a low-chlorophyll diet for 2 weeks to reduce auto-fluorescence in the intestinal region [27] and the abdominal hair was removed. Mice were anesthetized with isofluorane and received 200 μL Gd-Cy5.5 intravenously (dose of 500 mg/kg), and the accumulation of Cy5.5 into the urinary bladder was followed over time.

**Figure 5:**
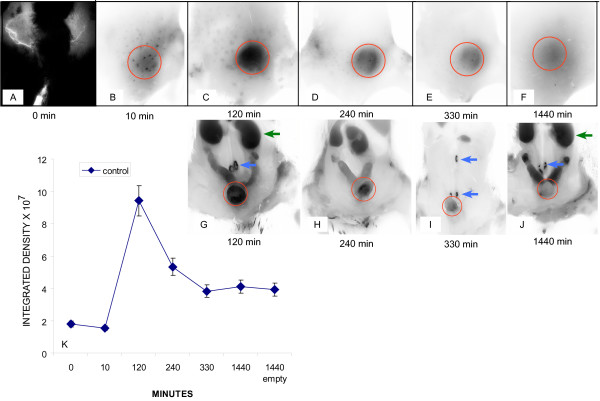
**Lymphatic Vessel Function (LVF) by NIRF**. For NIRF, a total of 9 SV40-*lacZ *mice ages 6–11 months were used. Mice were fed a low-chlorophyll diet for 2 weeks to reduce auto-fluorescence in the intestinal region and the abdominal hair was removed. Mice were anesthetized with isofluorane and received 200 μL Gd-Cy5.5 intravenously (dose of 500 mg/kg), and the accumulation of Cy5.5 into the urinary bladder was followed over time (**A – F)**. Additional mice, represented in **G-J**, have their abdomen opened and the gastrointestinal tract removed to permit better visualization of the pelvic floor. NIRF of the regions within red circles on **A-F **is represented as average ± SEM of the integrated density × 10^7 ^(**K)**. The numbers in each segment indicate the time lapse after i.v. administration of Gd-Cy5.5. Outside the urinary bladder, Cy5.5 accumulated in the kidneys (green arrows) and in lymph sacs (blue arrows in **G, I, and J**).

A time-course of Cy5.5 accumulation in the lower abdominal region of SV40-*lacZ *is illustrated in Figures 5A–5F. Additional mice, represented in Figures 5G–J, have their abdomen opened and the gastrointestinal tract removed to permit better visualization of the pelvic floor. Near infra red fluorescence of the regions within red circles on Figures 5A–F is represented as integrated density in Figure 5K. The numbers in each segment indicate the time lapse after i.v. administration of Gd-Cy5.5. An absence of specific fluorescence in the lower abdominal regions of mice at zero and 10 min after Gd-Cy5.5 administration (Figure 5A and 5B) is observed. A gradual accumulation of Cy5.5 was observed and a fluorescence peak occurred in the bladder region at 120 minutes (Figure 5B and 5F). At 240 minutes a significant amount of fluorescence still remained in the urinary bladder (Figure 5C and 5G). At 330 minutes the urinary bladders revealed low NIRF (Figure 5E and 5I) when compared to the fluorescence peak observed at 120 minutes, and at 24 hours (1440 min) only residual fluorescence was observed (Figure 5J). In addition, it should be noted that emptying the residual urinary content of the bladder did not alter significantly the intensity of fluorescence, indicating that the residual fluorescence corresponded to Cy5.5 which still remained within the bladder parenchyma. Outside the urinary bladder, Cy5.5 accumulated in the kidneys (green arrows) and in lymph sacs (blue arrows in Figures 5G, 5I and 5J).

### Co-localization of Cy5.5 with LYVE-1 positive lymphatics and CD31-positive blood vessels

**Figure 6:**
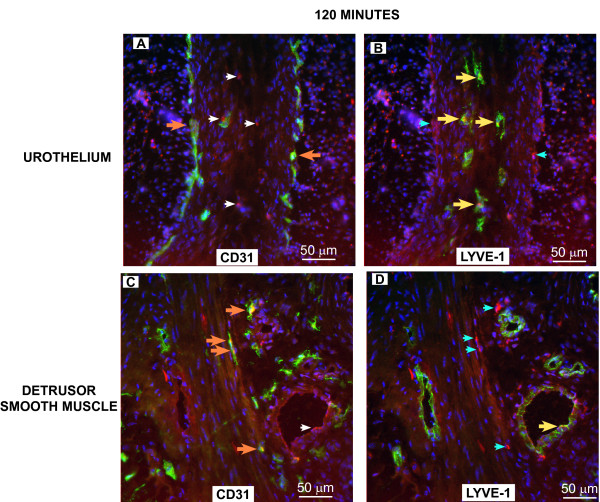
**Distribution of Cy5.5 between blood and lymphatic vessels at 120 minutes after administration of Gd-Cy5.5**. To confirm the results obtained *in vivo*, groups of mice (n = 3) were euthanized at 120 minutes after i.v. administration of Gd-Cy5.5 and the bladders were removed, frozen, and prepared for confocal analysis of the co-localization of Cy5.5 with CD31-positive vessels (green fluorescence in **A **and **C**) or LYVE-1-positive lymphatic vessels (green fluorescence in **B **and **D**). **A **and **B **are representative microphotographs of the same sub-epithelial region, whereas **C **and **D **are representative photomicrographs of the detrusor smooth muscle. Note that blood vessels are just underneath the urothelial cell layer **(A), **whereas lymphatic vessels are deeper in the submucosa **(B)**. The white arrows in **A **indicate Cy5.5 (red) that is co-localized with LYVE-1-positive cells in **B **(yellow arrows). The cyan arrow in **B** indicates Cy5.5 (red) that is co-localized with CD31-positive blood vessels in **A** (orange arrows). In all figures, the red represents Cy5.5 accumulation and the blue stain is DAPI. The yellow fluorescence indicates co-localization of Cy5.5 with CD31-positive blood vessels (**A**) or LYVE-1-positive lymphatic vessels (**B**). Quantification of the fluorescent signals by image analysis is presented in **Figure 9**.

**Figure 7:**
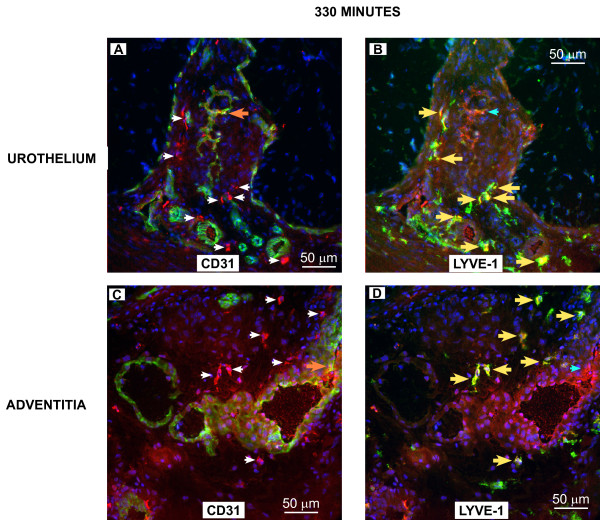
**Distribution of Cy5.5 between blood and lymphatic vessels at 330 minutes after administration of Gd-Cy5.5**. Photomicrographs obtained 330 minutes after i.v. administration of Gd-Cy5.5. **A **and **B **are representatives of the urothelial region whereas **C **and **D **are representatives of the adventitial region. Arrow's color scheme, as described in Figure 6. Quantification of the fluorescent signals by image analysis is presented in **Figure 9**.

**Figure 8:**
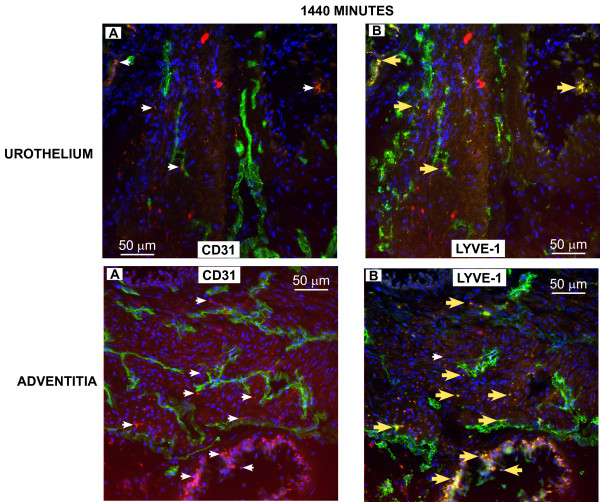
**Distribution of Cy5.5 between blood and lymphatic vessels at 1440 minutes after administration of Gd-Cy5.5**. Photomicrographs obtained 1440 minutes after i.v. administration of Gd-Cy5.5. **A **and **B **are representatives of the urothelial region, whereas **C **and **D **are representatives of the adventitial region. Arrow's color scheme, as described in **Figure 6**. Quantification of the fluorescent signals by image analysis is presented in **Figure 9**.

**Figure 9:**
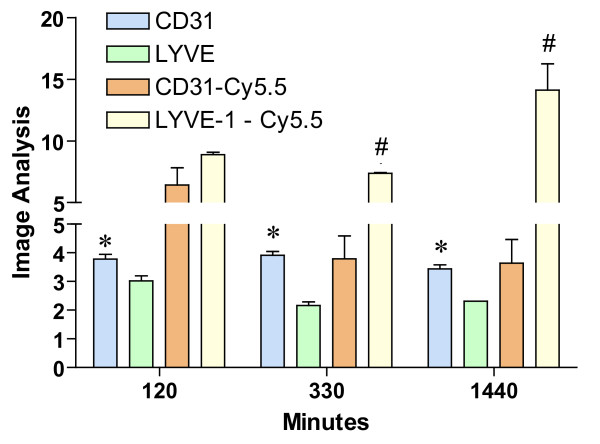
Image Analysis of bladder cross-sections obtained at 120, 330, and 1440 minutes (n = 3 mice per time point). Six random fields per cross-section were visualized at 20× magnification and used for image analysis that was performed with the NIS-Elements Advanced Research 2.3 imaging software (see Methods). Results are presented as mean and SEM. Asterisks indicate a statistically significant difference (p < 0.05) between the bladder area covered by blood vessels (CD-31) and lymphatic vessels (LYVE-1), whereas a pound sign indicates a statistically significant difference (p < 0.05) between the distribution of Cy5.5 in CD-31-positive blood vessels and LYVE-1-positive lymphatic vessels.

To confirm the results obtained *in vivo*, groups of mice (n = 3) were euthanized at 2, 5.5, and 24 hours (120, 330, and 1440 minutes) after i.v. administration of Gd-Cy5.5 and the bladders were removed for analysis of co-localization of Cy5.5 with LYVE-1- and CD31-positive cells. Figures 6, 7, 8 contain representative photomicrographics of confocal analysis of bladder cross-sections and the results of image analysis performed in all sections are presented in Figure 9. In all figures, the red represents Cy5.5 accumulation and the blue stain is DAPI (4',6-diamidino-2-phenylindole), which highlights the cell *nuclei*. The yellow label indicates co-localization of Cy5.5 with CD31-positive blood vessels, or LYVE-1-positive lymphatic vessels.

In particular Figures 6 A–B and 7 A–B indicate the sub-urothelial localization of blood vessels whereas lymphatic vessels are seen in the deeper regions of the mucosa but not in the subepithelial layer. Figures 8 A–D indicate that, at 1140 minutes, a significant amount of Cy5.5 still remains in the bladder parenchyma which may explain the fluorescence observed in Figure 5K.

Image analysis indicates that at all time points the area of the bladder cross-sections occupied by CD31-positive blood vessels was greater than the area occupied by LYVE-1-positive lymphatic vessels (Figure 9). In terms of co-localization of Cy5.5, at 120 minutes, Cy5.5 was found evenly distributed between LYVE-1-positive lymphatics and CD31-positive blood vessels (Figure 9). At 330 minutes a great proportion of Cy5.5 was found within LYVE-positive lymphatic vessels, and at 1140 minutes, most of the Cy5.5 was found within lymphatic vessels (Figures 9). These results provided the basis for subsequent MRI analysis of lymphatic vessel function.

### NIRF visualization of abdominal large lymphatic vessel draining the urinary bladder

**Figure 10:**
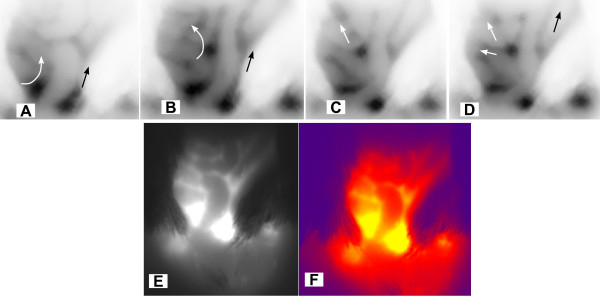
**NIRF visualization of abdominal large lymphatic vessel draining the urinary bladder**. A constant feature observed following i.v. administration of Cy5.5-conjugates was the labeling of abdominal lymphatic sacs (blue arrows in **Figures 5G, 5I, and 5J**). In order to investigate whether NIRF would permit visualization of these collecting lymphatic vessels and lymph sacs, high molecular weight dextran (500,000 MW)-Cy5.5 was injected in the pelvic floor underneath the urinary bladder, in the same region of lymph sacs of anesthetized mice, and serial images were immediately captured. **Figures 10 A-D **are negative NIRF images acquired along with time and indicate the movement of Cy5.5 in major collecting lymphatic vessels. It was noted that a series of lymph sacs were observed squirting the fluorescent content rhythmically and upwards (black and white arrows indicate the direction of the flow in two major collecting lymphatic vessels). A quick time movie, **Figure 10 E **illustrates the upward movement of Cy5.5 within collecting lymphatic vessels. **Figures 10 F **and 10 **G **are a high magnification of an individual frame of the movie illustrated in Figure 11 and additional file 1.

**Figure 11:**
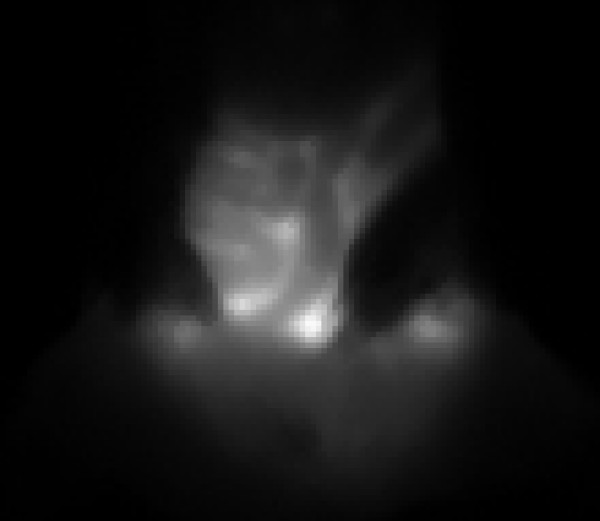
Isolated frame of a movie (additional file 1) presenting the visualization of high molecular weight dextran (500,000 MW)-Cy5.5 by NIRF.

A constant feature observed following i.v. administration of Cy5.5-conjugates was the labeling of abdominal lymphatic sacs (blue arrows in Figures 5G, 5I, and 5J). In order to investigate whether NIRF would permit visualization of these collecting lymphatic vessels and lymph sacs, high molecular weight dextran (500,000 MW)-Cy5.5 was injected in the pelvic floor underneath the urinary bladder, in the same region of lymph sacs of anesthetized mice and serial images were immediately captured. Figures 10 A–D are negative NIRF images acquired along with time and indicate the movement of Cy5.5 in major collecting lymphatic vessels and Figures 10E and 10F are higher magnifications of the same system. It was noted that a series of lymph sacs were observed squirting the fluorescent content rhythmically and upwards (black and white arrows indicate the direction of the flow in two major collecting lymphatic vessels). A quick time movie (Additional File 1), illustrates the upward movement of Cy5.5 within collecting lymphatic vessels shown in Figure 11.

**Figure 12:**
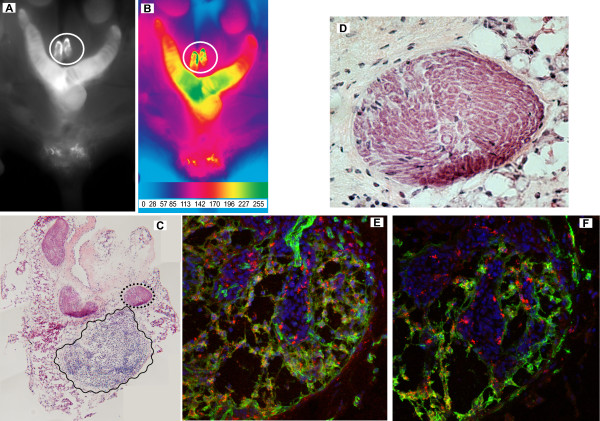
**NIRF-guided capture of lymph sacs**. Dextran-500,000 MW-Cy5.5 was administered in the pelvic floor underneath the urinary bladder. Two hours after dextran-Cy5.5, the abdomen was opened and the gastrointestinal tract was removed, and representative photomicrographs were taken in black-and-white (**A**) or artificially-colored to provide a gradation of fluorescence distribution (**B**). Fluorescent lymph sacs (white circle in **A **and **B**) were captured and dissected under fluorescence, and subsequently frozen for histology. **C **is a composite of an H&E stained cross-section (originally at ×100) of a lymph sac showing an interesting pattern of concentric paths of smooth muscle (dotted black circle) that can be appreciated at higher magnification in **D**. A region with high concentration of lymphocytes was also visible (wavy black line in figure **C**). This region was processed for fluorescent Immunohistochemistry and at the confocal level, it was possible to determine the presence of CD-31-positive blood vessels (**E**; green) and LYVE-1-positive lymphatic vessels (**F**; green) draining this region. In figures **E **and **F**, the red represents Cy5.5 accumulation and the blue stain is DAPI. Original magnifications were: **C **= ×100, **D **= ×400, **E **= ×400, and **F **= ×400.

Two hours after dextran-Cy5.5, the abdomen was opened and the gastrointestinal tract was removed, and representative photomicrographs were taken in black-and-white (Figure 12A) or artificially-colored to provide a gradation of fluorescence distribution (Figure 12B). It can be noticed that Cy5.5 accumulation occurred primarily in the bladder, uterus, and lymph sac or cysterna. NIRF also permitted the guided capture of tissues or organs with high fluorescence intensity. In this way, lymph sacs were dissected under fluorescence and processed for histology. Figure 12C is a composite of a cross-section of a lymph sac showing an interesting pattern of concentric paths of smooth muscle (black dotted circle) that can be appreciated at higher magnification in Figure 12D. A region with high concentration of lymphocytes was also visible (wavy black line on Figure 12C) and at the confocal level, it was possible to determine the presence of CD-31-positive blood vessels (Figure 12E; green) and LYVE-1-positive lymphatic vessels (Figure 12F; green) draining this region. In both figures, the red represents Cy5.5 accumulation and the blue stain is DAPI.

Although this work is very preliminary, it permitted our laboratory to identify and measure lymphatic function *in vivo *and in real time. These results indicate that NIRF can be use to study how Cy5.5-conjugate compounds are distributed between the interstitial fluid, blood vessels, and lymphatics. Figure 5K illustrates the kinetics of Gd-Cy5.5 and permitted the follow up of lymphatic vessel function by MRI (see below). In addition, large collecting lymphatics can be identified, the propulsion of lymph within lymphatic vessels can be visualized, and movies can be used to study these dynamics (Figure 11). Finally, NIRF-guided capture permitted us to start observing collecting lymphatics and lymph sacs and, therefore, to further study their structures.

### Magnetic Resonance Imaging of mouse urinary bladder cancer

Thanks to a unique rodent-dedicated MRI facility on campus [34], we were able to meet two major goals regarding the SV40-*lacZ *mice. The first goal was to detect the presence of BC as early as possible. This permitted a longitudinal study of tumor sizes during cancer progression. The second goal was to determine whether the observed increase in LVD was accompanied by an increased lymphatic function. A total of 7 SV40-*lacZ *mice and 3 wild type controls (FVB crossed with κB-*lacZ *mice) entered the study. Out of the 7 SV40-*lacZ *mice, 5 developed bladder tumors and 1 had to be euthanized.

**Figure 13:**
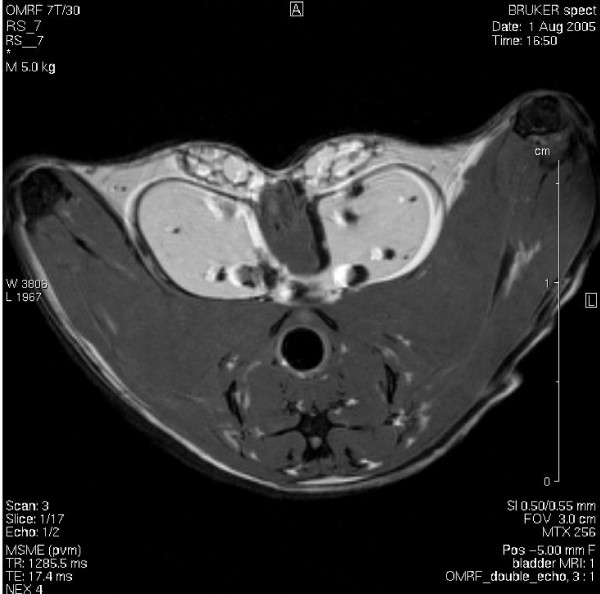
**Double echo pre-contrast MRI**. This is a representative frame of a double echo MRI of 6-month old SV40-*lacZ *mouse bladder indicating the different scanning positions. A movie is illustrated in additional file 2.

**Figure 14:**
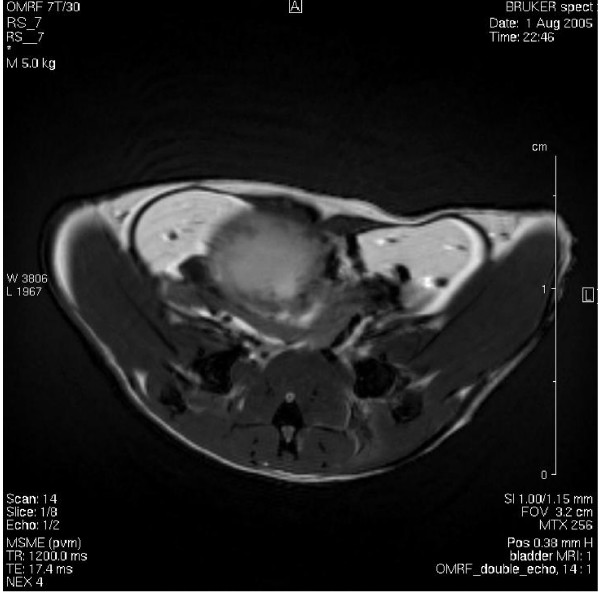
**Double echo post-contrast MRI**. This is a representative frame of a double echo MRI obtained in the same mouse bladder represented in Figure 13 after injection of Gd-Cy5.5. A movie is illustrated in additional file 3.

**Figure 15:**
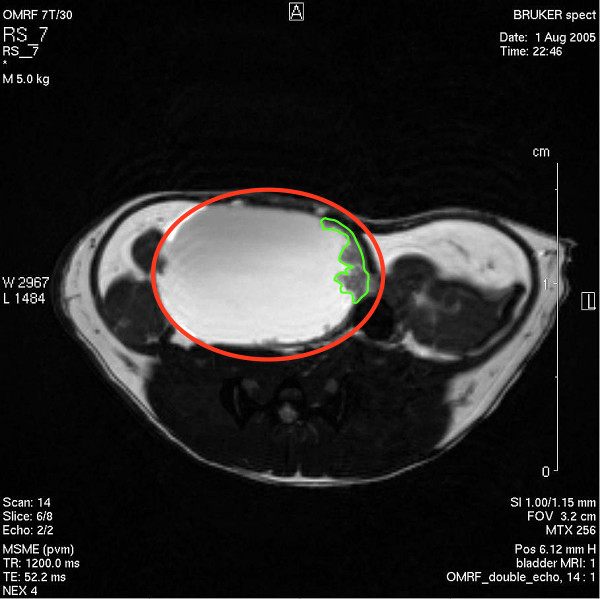
Detail of the last frame of movie presented in Figure 14 (additional file 3) indicating the urinary bladder (red circle) and tumor areas that are darker than the normal tissue (waved green line delimit tumor areas).

**Figure 16:**
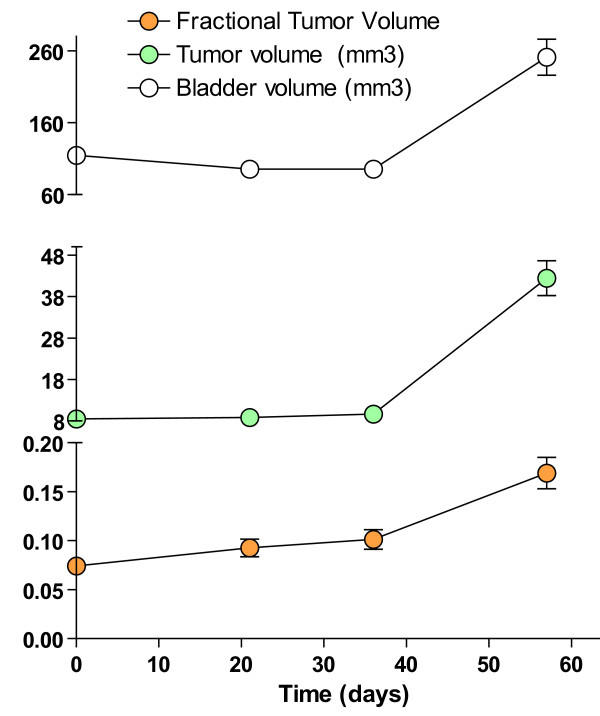
Longitudinal studies of tumor size during the period of 2 months (n = 5). Mean and SEM values for tumor volume, bladder volume, and fractional tumor volume obtained from five SV40-*lacZ *mice developing bladder tumor.

We were successful in early bladder cancer detection, and MRI technology permitted the detection of bladder cancer as early as in 6 months (youngest animal scanned). Figures 13, 14, and 15 are representative double echo MRIs of 6-month old SV40-*lacZ *mouse bladder. Additional file 2 is a movie obtained during pre-contrast MRI indicating the different scanning positions (Figure 13). Additional file 3 is a post-contrast (Gd-Cy5.5) MRI movie that is highlighted in Figure 14. Figure 15 is a detail of the last frame of movie presented in additional file 3 (a red circle delimits the urinary bladder and waved green line delimits tumor areas that are darker than the normal tissue). Overall, the urinary bladders averaged volumes of 219.2 ± 74 mm^3^. In contrast, the total area of the tumors averaged 0.31 ± 0.1 cm^2^, occupying areas of 31 ± 12.7 mm^2 ^and volumes of 15.6 ± 6 mm^3^. The smallest bladder tumor detected by MRI had a volume of 2.1 mm^3^. The tumor fractional volume in comparison with the whole bladder averaged 0.11 ± 0.02%. Longitudinal studies indicated that in some mice the tumor tripled its initial volume over 2 months (Figure 16).

### Dynamic contrast-enhanced MRI and Gd-Cy5.5 for determination of LVF

**Figure 17:**
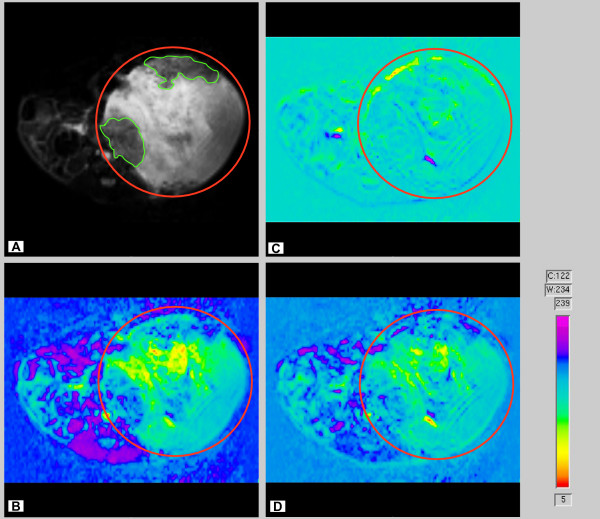
**MRI of mouse bladder using Gd-Cy5.5 as contrast agent**. **A**. Gray-scale image of the bladder and tumors at pre-contrast before Gd-probe administration. **B**. Difference image obtained at 30 minutes post-Gd-probe contrast subtracted from the pre-contrast image. This image demonstrates an early enhancement by the contrast agent into the urinary bladder extravascular space, which may be attributed to vascular leakage. **C**. Difference image obtained at 80 minutes post-contrast subtracted from 30 minutes post-contrast. This image demonstrates a delayed enhancement due to drainage and pooling of the contrast agent, which may reflect uptake within the lymphatics in the bladder. **D**. Difference image obtained between images in **B **and **C **(80 min. post-contrast minus 30 min post-contrast, and the 30 min. post-contrast minus the pre-contrast images). This image suggests that there is decreased drainage within tumorigenic areas, and perhaps a pronounced increase in uptake in the lymphatics within normal regions.

Dynamic contrast-enhanced MRI (DCE-MRI) images with the use of the contrast agent Gd-Cy5.5 were acquired subsequent to anatomical scans providing sensitive regions of interest (ROI) for calculating T1 values between normal and bladder tumor relaxation times. We found an increased signal (due to decreased T1 relaxation) that correlated with an increased uptake of the Gd-probe from the blood vessels into the urinary bladder extra vascular space. The bladder volume did not change significantly during the imaging period. However, there may have been some motion artifacts associated with respiratory motion that was not compensated with respiratory gating. Figure 17A is a gray scale representation of peak accumulation of the Gd-probe in the bladder wall, except within tiny regions (a red circle delimits the urinary bladder and green lines indicated two tumor areas that are darker than the normal tissue). Figure 17B represents the difference image obtained at 30 minutes post-Gd-probe contrast subtracted from the pre-contrast image. This image illustrates an early enhancement by the contrast agent into the urinary bladder extravascular space, which may be attributed to vascular leakage. Figure 17C represents difference image obtained at 80 minutes post-contrast subtracted from 30 minutes post-contrast. This image demonstrates a delayed enhancement due to drainage and pooling of the contrast agent, which may reflect uptake within the lymphatics in the bladder. Figure 17D represents the difference image obtained between images in 17B and 17C (80 min. post-contrast minus 30 min post-contrast, and the 30 min. post-contrast minus the pre-contrast images). This image suggests that there is decreased drainage within tumorigenic areas and perhaps a pronounced increase in uptake in the lymphatics within normal regions.

**Figure 18:**
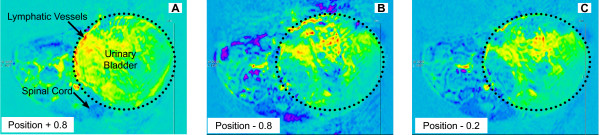
**Digital subtraction of the images between pre- and 80 minutes post-contrast**. Images were acquired in two "phases" corresponding to the biphasic kinetics of the BSA-Gd-DTPA [15]. The first or "early phase" comprised of images obtained just before i.v. administration of 200 μL Gd-Cy5.5 (dose of 500 mg/kg). Starting at 3 minutes post-injection, images were acquired every 7 minutes, up to 2 hours post-contrast. The second block of MRI data was acquired up to 80 minutes after the first series of images. Images represents accumulation of Gd-Cy5.5 represented in three contiguous slices from the same bladder (positions -0.2 = A, -0.8 = B, and +0.8 = C). The red and yellow regions are active lymphatic vessels and consist of drainage events within the bladder interstitium.

**Figure 19:**
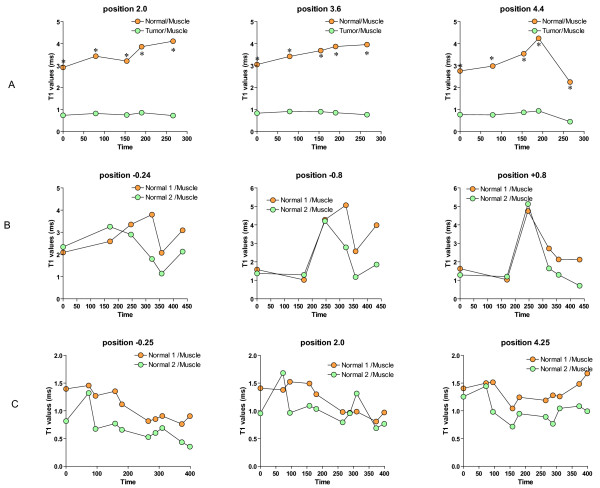
Time-dependent clearance of the Gd-probe agent away from the bladder provides a strong indication of lymphatic vessel function (LVF). **Figure 18 **represents the comparison between tumor-bearing bladders (**Figure A**) and controls (**Figures B and C**). Data represents T1 values in [ms] with Bruker's MSMEVTR spin-echo method, including 3 slice packages chosen to include both normal and tumor regions in bladder. T1 values from these regions were normalized as percent of the reference site (muscle). In control bladders, two different normal regions were compared. This figure represents the time-dependent clearance of the contrast agent away from the bladder and provides a strong indication of LVF. Asterisks indicate a statistical significant difference (p < 0.05) between normal and tumor areas.

Because drainage of macromolecules by the lymphatics is a slow event with rates of 2.3 to 3.7 μl/g minutes, images were acquired in two "phases" corresponding to the biphasic kinetics of the BSA-Gd-DTPA [15]. The first or "early phase" is comprised of images obtained just before i.v. administration of 200 μL Gd-Cy5.5 (dose of 500 mg/kg). Starting at 3 minutes post-injection, images were acquired every 7 minutes, up to 2 hours post-contrast. The second block of MRI data was acquired up to 80 minutes after the first series of images. A digital subtraction of the images between pre- and 80 minutes post-contrast following Gd-Cy5.5 in three contiguous slices from the same bladder (positions -0.2, -0.8, and +0.8) was artificially colored (Figures 18A, 18B, and 18C). The red and yellow regions are active lymphatic vessels and consist of drainage events within the bladder interstitium. Figure 19 represents the comparison between tumor-bearing bladders (Figure 19A) and controls (Figure 19B and 19C). T1 values from these regions were normalized as percent of the reference site (muscle). This figure represents the time-dependent clearance of the contrast agent away from the bladder and provides a strong indication of LVF. Note the difference in T1 values between a tumor region and an adjacent normal bladder region (Figure 19A). In control bladders, two different normal regions were compared. These results suggested decreased drainage in the areas bearing tumors in comparison to normal areas.

## Discussion

Our results with SV40-*lacZ *mice indicate that in the normal bladder, a rich lymphatic vessel network is visible from the adventitia through the detrusor smooth muscle. It is characterized as a vascular network of blind ended, thin-walled capillaries that merge to larger collecting ducts, all positively stained with LYVE-1 antibody. In sharp contrast with the dense blood vessel vascularization of the bladder mucosa, we found that lymphatic vessels are absent of the sub-urothelium being located much deeper in the lamina propria (Figures 6A, 6B, 7A, and 7B) which seems to parallel the clinical findings in the human bladder [35,36].

Our analysis indicates an increased lymphatic density during cancer progression and a co-localization of Ki-67 with some of LYVE-1-positive lymphatics, suggesting cancer-induced lymphangiogenesis (Figure 4), as it was recently suggested in human bladder cancer [10,37]. However, the mechanisms for bladder lymphangiogenesis are not clear. In contrast to blood vessel angiogenesis, the mechanisms of lymphangiogenesis in general are still relatively vague [38]. Vascular endothelial growth factor-C (VEGF-C) and VEGF-D have been implicated as specific regulators of lymphangiogenesis [39-43]. Both growth factors mediate their biological activity mainly by VEGF receptor-3 (VEGFR-3, Flt-4) [44,45]. It remains to be determined whether VEGF-C and VEGF-D along with VEGFR-3 play a role in lymphangiogenesis during bladder cancer development. Another interesting line of research involves LYVE-1-positive tumor associated macrophages [46] that have indeed been associated with tumor lymphangiogenesis [47,48]. In the eye, a stepwise mechanism of inflammation-associated *de novo *lymphangiogenesis involves potential lymphatic progenitor cells [49] derived from circulation that transmigrate through the connective tissue stroma, presumably in the form of macrophages [49-51], and finally incorporate into the growing lymphatic vessels [52]. Our present findings indicate that in addition to LYVE-1-positive lymphatic endothelial cells, during bladder cancer development, LYVE-1 positive macrophages were found in the bladder detrusor muscle isolated from SV40-*lacZ *mice. Although this may be only a circumstantial finding, it opens a testable hypothesis on the role of macrophages and other inflammatory cells in bladder lymphangiogenesis. The introduction of SV40-*lacZ *along with the visualization and quantification techniques described here will permit further investigation on this subject.

It has been proposed that lymphangiogenesis is correlated with tumor metastasis. Increasing knowledge of the tumor's biological significance in lymphatics within the tumors (intratumoral lymphatics, ITLs) and at the tumor periphery (peritumoral lymphatics, PTLs) has greatly promoted understanding of tumor access into the lymphatic system by inducing lymphangiogenesis or by co-opting preexisting lymphatics [2]. Indeed, peritumoral lymphatics have also been associated with both regional metastasis and survival in bladder [37], lung [53], breast [54], and prostate cancer [55]. But the question still remains as to whether pre-existing vessels are sufficient to serve this function, or whether tumor cell dissemination requires *de novo *lymphatic formation or an increase in lymphatic size. In this regard, Fernandez and collaborators reported that in the human bladder, higher intratumoral LVD correlates significantly with poor histological differentiation, and that higher peritumoral LVD showed a significant association with the presence of lymph node metastasis [10]. Although peritumoral lymphatic vessels contribute to tumor metastasis, opposite views exist as to whether intratumoral lymphatics have any role in tumor metastasis [56,57]. Padera and collaborators examined functional lymphatics associated with mouse tumors expressing normal or elevated levels of VEGF-C [58]. Although VEGF-C over-expression increased lymphatic surface area in the tumor margin and lymphatic metastasis, these tumors contained no functional lymphatics, as assessed by four independent functional assays and IHC staining [58]. These findings suggest that the functional lymphatics in the tumor margin alone are sufficient for lymphatic metastasis and should be targeted therapeutically [58]. Our MRI results are in agreement with those described by Pandera and collaborators [58] in the sense that intratumor lymphatic vessels have a reduced function when compared to normal areas of the bladder.

Another question answered by the present work was whether an increase in the number of lymphatic vessels leads to increased function. For this purpose, we followed the strategy recently reviewed by Neeman and collaborators [59]. The contrast agent introduced here was originally described by Dafni and collaborators [14-16], and subsequently by Pathak and collaborators [15], for visualization of lymphatics and determination of their function. The modification of conjugating biotin-BSA-Gd-DTPA [15] to Cy5.5 permitted us to use NIRF and MRI to follow the dynamics of the same compound and calculate lymphatic vessel function. The advantage of using NIRF is the faster time for data acquisition. NIRF information permitted us to narrow the number of time points for subsequent MRI studies.

The proposed mechanism of visualization of BSA-Gd-Cy5.5 (~82 kDa) is based on the presence of BSA which prolongs its lifetime in circulation. Initially the probe is confined to blood vessels and it is systemically distributed, and as the time passes it extravasates to the extracellular space in points of increased vascular permeability. Both NIRF and MRI are able to detect when BSA-Gd-cy5.5 starts to accumulate in the extracellular space and when its accumulation reaches a plateau. This was done at the MRI level by following the longitudinal relaxation rates (1/T1) that correlates with increased uptake of the Gd-probe from the blood vessels into the urinary bladder extra vascular space. This phase was called the "early phase" in this manuscript. After 80 minutes of the plateau, a second series of MRI were started and followed the clearance of BSA- Gd-Cy5.5 from the extravascular by the lymphatic vessels. This was supported by *ex vivo *images which indicated that, at this point, most of the BSA-Gd-Cy5.5 is drained from the tissue by lymphatic vessels.

Although MRI lymphangiography does measure areas of draining and pooling instead of directly evaluating lymphatic vessel function, the delayed enhancement observed at later time points from the Gd-probe may also reflect uptake of Gd-probe in the lymphatics. In addition, this information can not be obtained any other way and clinical studies attest the validity of using lymphangiography for assessment of lymphatic vessel function [60-64]. A step forward in this direction was introduced here by the use of a single probe that can be imaged by MRI and NIRF. The first indicates areas of draining and pooling and the second permitted the temporal association of images with cross-sections indicating the relative distribution of the probe between CD31-positive blood vessels and LYVE-1-positive lymphatic vessels.

As pointed out by Neeman and collaborators [59], contrast MRI data are typically dominated by vascular permeability, which often masks the relatively slow lymphatic drain. To separate vascular leakage from the lymphatic drain, an avidin chase needs to be introduced [14,16] which through the rapid clearance of intravenously administered biotin-BSA-Gd-DTPA, allowed them to experimentally track interstitial convection and lymphatic drain in the absence of continuing vascular leakage [14,16]. Our present MRI results are not corrected for vascular leakage. Therefore, future experiments will take into consideration this point for a more detailed analysis of lymphatic vessel function using the advantage of the presence of biotin in the Gd-Cy5.5 molecule.

Another advantage of NIRF was to permit the collection of tissues exhibiting fluorescence. In this regard, we were able to further pursue the morphology of lymph sacs using dextran-Cy5.5. This will allow us in the near future to determine the contractility of lymph sacs and abdominal lymphatic vessels. The rhythmic activity observed in the abdominal lymphatics draining the urinary bladder is in agreement with recent results that indicated that mesenteric lymphatic vessels have a true pacemaker mechanism [65,66]. The experiment described in Figures 10, 11, 12 will permit the capture and evaluation of large lymphatic function in health and disease.

## Conclusion

The present work introduces a new double transgenic mouse model that permits the visualization of lymphatic vessels during bladder cancer progression, and introduces new technologies for the visualization and quantification of lymphatic vessel density and function by combining NIRF and MRI imaging. The results presented here raise the possibility of the study of lymphatic vessel activity *in vivo *and in real time and raises the hypothesis regarding the role of lymphangiogenesis during bladder cancer progression. It remains to be determined whether manipulation of lymphatic vessel density and function would alter bladder tumor progression.

## Abbreviations

IHC, immunohistochemistry; MRI, magnetic resonance imaging; and NIRF, near infrared fluorescence.

## Competing interests

The author(s) declare that they have no competing interests.

## Authors' contributions

All authors read and approved the final manuscript. **MRS **conceived study and experimental design, participated in NIRF experiments, performed IHC analysis, interpreted the results, and drafted the manuscript; **RT **performed MRI experiments, participated on the design, results interpretation, and helped drafting the manuscript; **NS **synthesized the d-Cy5.5; **AA **performed MRI experiments and interpretation of MRI results; **MN **consulted **MRS **regarding the proper contrast agents, participated in the design of MRI experiments and interpretation of the results, and helped drafting the manuscript; **CAD **maintained the genotyping and animal colony, and performed image analysis; **CS **performed animal experiments and whole mount IHC; **JM **participated on the design of immunohistochemistry protocols and choice of antibodies, performed the immunohistochemistry and confocal microscopy analysis; **SM **developed kB-lacZ mice, participate in the design of the double transgenic, and helped drafting the manuscript; **X-RW **developed UPKII-SV40T mice, participate in the design of the double transgenic, and helped drafting the manuscript; and **RS** participated in the experimental design, performed NIRF experiments and data interpretation.

## Pre-publication history

The pre-publication history for this paper can be accessed here:



## Supplementary Material

Additional File 1 Large lymphatics draining the lower urinary tract. Quick time movie presenting the visualization of high molecular weight dextran (500,000 MW)-Cy5.5 by NIRF injected in large collecting lymphatics draining the lower urinary tract. Click here for file

Additional File 2 **Movie obtained during pre-contrast MRI**. This is a representative double echo MRI of 6-month old SV40-*lacZ *mouse bladder indicating the different scanning positions. Click here for file

Additional File 3 **Movie obtained post-contrast MRI**. This is a representative double echo MRI obtained in the same mouse bladder represented in Figure 14 after injection of Gd-Cy5.5. Click here for file
